# 1-Methyl-1*H*-indazole-3-carboxylic acid

**DOI:** 10.1107/S160053680803554X

**Published:** 2008-11-08

**Authors:** Si-shun Kang, Hong-wei Wang, Min Zhang, Ran-zhe Lu, Hai-bo Wang

**Affiliations:** a College of Science, Nanjing University of Technology, Xinmofan Road No. 5 Nanjing, Nanjing 210009, People’s Republic of China; b Nantong Entry–Exit Inspection and Quarantine Bureau, Nantong 226005, People’s Republic of China

## Abstract

The asymmetric unit of the title compound, C_9_H_8_N_2_O_2_, contains two mol­ecules. In the crystal structure, both mol­ecules form inversion dimers *via* pairs of O—H⋯O hydrogen bonds, and a C—H⋯O inter­ation is also seen.

## Related literature

For the synthesis, see: Rousseau & Lindwall (1950 ▶).
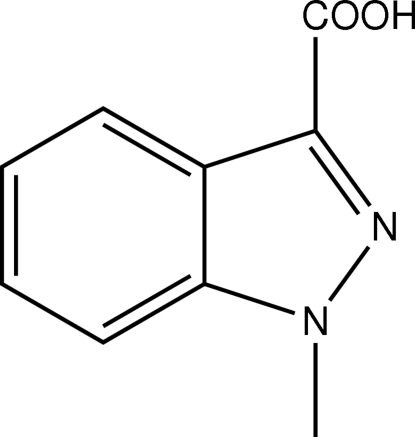


## Experimental

### 

#### Crystal data

C_9_H_8_N_2_O_2_
*M* *_r_* = 176.17
Monoclinic, 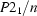

*a* = 7.5470 (15) Å
*b* = 14.873 (3) Å
*c* = 14.924 (3) Å
β = 93.10 (3)°
*V* = 1672.7 (6) Å^3^
*Z* = 8
Mo *K*α radiation
μ = 0.10 mm^−1^
*T* = 293 (2) K
0.30 × 0.20 × 0.10 mm

#### Data collection

Enraf–Nonius CAD-4 diffractometer
Absorption correction: ψ scan (North *et al.*, 1968 ▶) *T*_min_ = 0.970, *T*_max_ = 0.990
3273 measured reflections
3032 independent reflections
1955 reflections with *I* > 2σ(*I*)
*R*_int_ = 0.0225
3 standard reflections every 200 reflections intensity decay: 1%

#### Refinement

*R*[*F* ^2^ > 2σ(*F* ^2^)] = 0.058
*wR*(*F* ^2^) = 0.143
*S* = 1.00
3032 reflections
237 parameters
H-atom parameters constrained
Δρ_max_ = 0.18 e Å^−3^
Δρ_min_ = −0.21 e Å^−3^

### 

Data collection: *CAD-4 Software* (Enraf–Nonius, 1989 ▶); cell refinement: *CAD-4 Software*; data reduction: *XCAD4* (Harms & Wocadlo, 1995 ▶); program(s) used to solve structure: *SHELXS97* (Sheldrick, 2008 ▶); program(s) used to refine structure: *SHELXL97* (Sheldrick, 2008 ▶); molecular graphics: *SHELXTL* (Sheldrick, 2008 ▶); software used to prepare material for publication: *SHELXL97*.

## Supplementary Material

Crystal structure: contains datablocks global, I. DOI: 10.1107/S160053680803554X/hb2830sup1.cif

Structure factors: contains datablocks I. DOI: 10.1107/S160053680803554X/hb2830Isup2.hkl

Additional supplementary materials:  crystallographic information; 3D view; checkCIF report

## Figures and Tables

**Table 1:** Hydrogen-bond geometry (Å, °)

*D*—H⋯*A*	*D*—H	H⋯*A*	*D*⋯*A*	*D*—H⋯*A*
O2—H2*A*⋯O1^i^	0.82	1.82	2.632 (3)	173
O3—H3*A*⋯O4^ii^	0.82	1.82	2.619 (3)	164
C8—H8*A*⋯O1^iii^	0.93	2.52	3.293 (4)	140

Symmetry codes: (i) 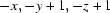; (ii) 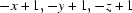; (iii) 
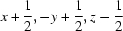
.

**Fig. 1.**
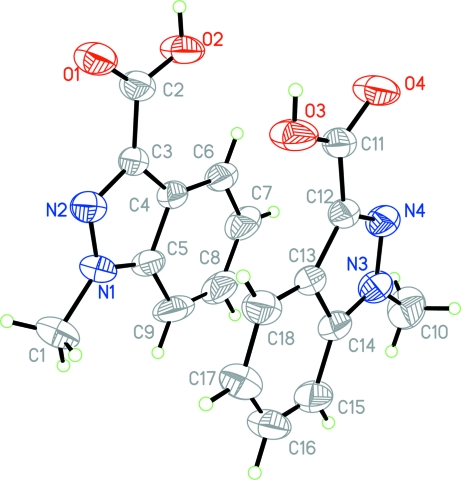
A view of the molecular structure of (I), showing displacement ellipsoids at the 30% probability level (arbitrary spheres for the H atoms).

## supplementary crystallographic information

### Comment

Methyl indazole carboxylic acid is an important pharmaceutical intermediate: many of its derivatives have biological activity and be used as a variety of drugs. We report here the crystal structure of the title compound, (I). There are O—H···O intermolecular H bonds in the structure between the hydrogencarboxylates forming the paired molecules that are situated on the crystallographic inversion centres (Table 1). The molecular structure of (I) is shown in Fig. 1.

### Experimental

We prepared the title compound according to the literature method (Rousseau & Lindwall, 1950). Colourless blocks of (I) were obtained by slow evaporation of an petroleum/metanhol solution.

### Refinement

The H atoms were placed geometrically (C—H = 0.93-0.97Å, O—H = 0.82Å) and refined as riding with *U*_iso_(H) = 1.2 or 1.5*U*_eq_(carrier).

### Crystal data

**Table:**

C_9_H_8_N_2_O_2_	*F*_000_ = 736
*M**_r_* = 176.17	*D*_x_ = 1.399 Mg m^−^^3^
Monoclinic, *P*2_1_/*n*	Mo *K*α radiation λ = 0.71073 Å
Hall symbol: -P 2yn	Cell parameters from 25 reflections
*a* = 7.5470 (15) Å	θ = 9–13º
*b* = 14.873 (3) Å	µ = 0.10 mm^−^^1^
*c* = 14.924 (3) Å	*T* = 293 (2) K
β = 93.10 (3)º	Block, colorless
*V* = 1672.7 (6) Å^3^	0.30 × 0.20 × 0.10 mm
*Z* = 8	

### Data collection

**Table:**

Enraf–Nonius CAD-4 diffractometer	*R*_int_ = 0.023
Radiation source: fine-focus sealed tube	θ_max_ = 25.3º
Monochromator: graphite	θ_min_ = 1.9º
*T* = 293(2) K	*h* = −9→9
ω/2θ scans	*k* = 0→17
Absorption correction: ψ scan(North *et al.*, 1968)	*l* = 0→17
*T*_min_ = 0.970, *T*_max_ = 0.990	3 standard reflections
3273 measured reflections	every 200 reflections
3032 independent reflections	intensity decay: 1%
1955 reflections with *I* > 2σ(*I*)	

### Refinement

**Table:**

Refinement on *F*^2^	Secondary atom site location: difference Fourier map
Least-squares matrix: full	Hydrogen site location: inferred from neighbouring sites
*R*[*F*^2^ > 2σ(*F*^2^)] = 0.058	H-atom parameters constrained
*wR*(*F*^2^) = 0.143	*w* = 1/[σ^2^(*F*_o_^2^) + (0.05*P*)^2^ + 1.2*P*] where *P* = (*F*_o_^2^ + 2*F*_c_^2^)/3
*S* = 1.00	(Δ/σ)_max_ = 0.007
3032 reflections	Δρ_max_ = 0.18 e Å^−^^3^
237 parameters	Δρ_min_ = −0.21 e Å^−^^3^
Primary atom site location: structure-invariant direct methods	Extinction correction: none

### Special details

**Table:**

Geometry. All e.s.d.'s (except the e.s.d. in the dihedral angle between two l.s. planes) are estimated using the full covariance matrix. The cell e.s.d.'s are taken into account individually in the estimation of e.s.d.'s in distances, angles and torsion angles; correlations between e.s.d.'s in cell parameters are only used when they are defined by crystal symmetry. An approximate (isotropic) treatment of cell e.s.d.'s is used for estimating e.s.d.'s involving l.s. planes.
Refinement. Refinement of *F*^2^ against ALL reflections. The weighted *R*-factor *wR* and goodness of fit *S* are based on *F*^2^, conventional *R*-factors *R* are based on *F*, with *F* set to zero for negative *F*^2^. The threshold expression of *F*^2^ > σ(*F*^2^) is used only for calculating *R*-factors(gt) *etc*. and is not relevant to the choice of reflections for refinement. *R*-factors based on *F*^2^ are statistically about twice as large as those based on *F*, and *R*- factors based on ALL data will be even larger.

### Fractional atomic coordinates and isotropic or equivalent isotropic displacement parameters (Å^2^)

**Table:**

	*x*	*y*	*z*	*U*_iso_*/*U*_eq_	
O1	−0.0113 (3)	0.38836 (12)	0.50834 (13)	0.0608 (6)	
O2	0.0844 (3)	0.46943 (12)	0.39536 (13)	0.0627 (6)	
H2A	0.0587	0.5109	0.4284	0.094*	
N1	0.0777 (3)	0.17493 (14)	0.34517 (17)	0.0511 (6)	
N2	0.0425 (3)	0.23313 (15)	0.41044 (16)	0.0491 (6)	
C1	0.0605 (5)	0.07913 (19)	0.3588 (3)	0.0738 (10)	
H1A	0.0634	0.0488	0.3021	0.111*	
H1B	−0.0500	0.0667	0.3852	0.111*	
H1C	0.1569	0.0583	0.3980	0.111*	
C2	0.0474 (4)	0.39448 (17)	0.43217 (19)	0.0451 (7)	
C3	0.0726 (3)	0.31492 (17)	0.37687 (17)	0.0402 (6)	
C4	0.1346 (3)	0.31004 (17)	0.28951 (17)	0.0398 (6)	
C5	0.1353 (3)	0.21700 (18)	0.27100 (19)	0.0444 (7)	
C6	0.1899 (3)	0.37047 (19)	0.22478 (18)	0.0478 (7)	
H6A	0.1896	0.4321	0.2355	0.057*	
C7	0.2442 (4)	0.3372 (2)	0.1459 (2)	0.0593 (8)	
H7A	0.2834	0.3769	0.1030	0.071*	
C8	0.2429 (4)	0.2443 (2)	0.1273 (2)	0.0620 (9)	
H8A	0.2790	0.2240	0.0722	0.074*	
C9	0.1899 (4)	0.1841 (2)	0.1883 (2)	0.0604 (9)	
H9A	0.1896	0.1228	0.1761	0.073*	
O3	0.4920 (3)	0.38818 (12)	0.47829 (12)	0.0587 (6)	
H3A	0.4752	0.4303	0.5122	0.088*	
O4	0.5840 (3)	0.50052 (13)	0.39334 (14)	0.0655 (6)	
N3	0.6825 (3)	0.30394 (16)	0.21444 (15)	0.0519 (6)	
N4	0.6574 (3)	0.37909 (15)	0.26119 (15)	0.0474 (6)	
C10	0.7390 (4)	0.3098 (2)	0.12273 (19)	0.0672 (9)	
H10A	0.7340	0.2512	0.0957	0.101*	
H10B	0.8584	0.3321	0.1234	0.101*	
H10C	0.6618	0.3499	0.0887	0.101*	
C11	0.5579 (3)	0.41736 (17)	0.40759 (18)	0.0433 (7)	
C12	0.5990 (3)	0.35246 (17)	0.33950 (17)	0.0380 (6)	
C13	0.5881 (3)	0.25681 (17)	0.34408 (18)	0.0406 (6)	
C14	0.6464 (4)	0.22852 (19)	0.26155 (19)	0.0465 (7)	
C15	0.6532 (4)	0.1370 (2)	0.2374 (2)	0.0616 (9)	
H15A	0.6879	0.1192	0.1813	0.074*	
C16	0.6078 (4)	0.0772 (2)	0.2984 (3)	0.0677 (10)	
H16A	0.6139	0.0163	0.2849	0.081*	
C17	0.5502 (4)	0.1039 (2)	0.3838 (2)	0.0669 (9)	
H17A	0.5181	0.0600	0.4243	0.080*	
C18	0.5410 (4)	0.19292 (18)	0.4077 (2)	0.0531 (7)	
H18A	0.5050	0.2101	0.4638	0.064*	

### Atomic displacement parameters (Å^2^)

**Table:**

	*U*^11^	*U*^22^	*U*^33^	*U*^12^	*U*^13^	*U*^23^
O1	0.1022 (16)	0.0363 (11)	0.0479 (12)	−0.0076 (11)	0.0402 (11)	−0.0043 (9)
O2	0.1051 (18)	0.0305 (11)	0.0568 (12)	0.0012 (11)	0.0438 (12)	−0.0025 (9)
N1	0.0550 (15)	0.0271 (12)	0.0718 (17)	0.0010 (10)	0.0096 (12)	−0.0088 (11)
N2	0.0551 (15)	0.0355 (13)	0.0581 (15)	0.0028 (11)	0.0157 (12)	−0.0012 (11)
C1	0.081 (2)	0.0279 (16)	0.114 (3)	0.0007 (16)	0.016 (2)	0.0012 (17)
C2	0.0571 (18)	0.0305 (15)	0.0490 (16)	0.0005 (13)	0.0142 (13)	0.0002 (12)
C3	0.0432 (15)	0.0302 (14)	0.0486 (15)	−0.0013 (12)	0.0164 (12)	−0.0019 (12)
C4	0.0373 (14)	0.0384 (15)	0.0442 (15)	0.0003 (12)	0.0076 (11)	−0.0119 (12)
C5	0.0376 (14)	0.0370 (15)	0.0588 (17)	0.0082 (12)	0.0053 (13)	−0.0111 (13)
C6	0.0505 (17)	0.0407 (16)	0.0540 (17)	0.0059 (13)	0.0190 (13)	−0.0018 (13)
C7	0.063 (2)	0.067 (2)	0.0506 (18)	0.0077 (16)	0.0274 (15)	−0.0083 (15)
C8	0.0560 (19)	0.072 (2)	0.060 (2)	0.0090 (17)	0.0216 (16)	−0.0223 (18)
C9	0.0556 (19)	0.0517 (19)	0.074 (2)	0.0187 (15)	0.0041 (16)	−0.0255 (17)
O3	0.0938 (16)	0.0357 (11)	0.0498 (12)	0.0046 (10)	0.0348 (11)	−0.0029 (9)
O4	0.1026 (17)	0.0302 (11)	0.0684 (14)	−0.0035 (11)	0.0474 (12)	−0.0045 (10)
N3	0.0545 (15)	0.0510 (15)	0.0530 (14)	−0.0023 (12)	0.0282 (11)	−0.0153 (12)
N4	0.0506 (14)	0.0439 (14)	0.0497 (14)	0.0008 (11)	0.0214 (11)	−0.0049 (11)
C10	0.070 (2)	0.085 (3)	0.0489 (18)	−0.0068 (19)	0.0272 (16)	−0.0100 (17)
C11	0.0487 (16)	0.0323 (15)	0.0505 (16)	−0.0016 (12)	0.0189 (13)	−0.0036 (12)
C12	0.0395 (15)	0.0341 (14)	0.0414 (14)	0.0003 (12)	0.0104 (11)	−0.0038 (12)
C13	0.0370 (14)	0.0360 (15)	0.0497 (16)	0.0003 (12)	0.0120 (12)	−0.0085 (12)
C14	0.0406 (15)	0.0433 (16)	0.0567 (18)	0.0041 (13)	0.0130 (13)	−0.0134 (14)
C15	0.0557 (19)	0.050 (2)	0.080 (2)	0.0011 (15)	0.0145 (16)	−0.0312 (18)
C16	0.065 (2)	0.0334 (17)	0.104 (3)	0.0039 (15)	0.000 (2)	−0.0169 (18)
C17	0.071 (2)	0.0361 (18)	0.094 (3)	0.0008 (16)	0.0151 (19)	0.0054 (17)
C18	0.0606 (19)	0.0383 (16)	0.0621 (18)	0.0027 (14)	0.0183 (15)	0.0017 (14)

### Geometric parameters (Å, °)

**Table:**

O1—C2	1.246 (3)	O3—C11	1.267 (3)
O2—C2	1.280 (3)	O3—H3A	0.8200
O2—H2A	0.8200	O4—C11	1.272 (3)
N1—N2	1.340 (3)	N3—N4	1.337 (3)
N1—C5	1.363 (4)	N3—C14	1.359 (4)
N1—C1	1.446 (3)	N3—C10	1.458 (3)
N2—C3	1.340 (3)	N4—C12	1.331 (3)
C1—H1A	0.9600	C10—H10A	0.9600
C1—H1B	0.9600	C10—H10B	0.9600
C1—H1C	0.9600	C10—H10C	0.9600
C2—C3	1.461 (4)	C11—C12	1.447 (3)
C3—C4	1.411 (3)	C12—C13	1.427 (4)
C4—C6	1.400 (4)	C13—C14	1.395 (4)
C4—C5	1.411 (3)	C13—C18	1.402 (4)
C5—C9	1.410 (4)	C14—C15	1.409 (4)
C6—C7	1.361 (4)	C15—C16	1.331 (5)
C6—H6A	0.9300	C15—H15A	0.9300
C7—C8	1.409 (4)	C16—C17	1.425 (5)
C7—H7A	0.9300	C16—H16A	0.9300
C8—C9	1.353 (4)	C17—C18	1.374 (4)
C8—H8A	0.9300	C17—H17A	0.9300
C9—H9A	0.9300	C18—H18A	0.9300
			
C2—O2—H2A	109.5	C11—O3—H3A	109.5
N2—N1—C5	112.2 (2)	N4—N3—C14	112.5 (2)
N2—N1—C1	120.8 (3)	N4—N3—C10	119.8 (2)
C5—N1—C1	127.0 (3)	C14—N3—C10	127.7 (2)
C3—N2—N1	105.7 (2)	C12—N4—N3	105.8 (2)
N1—C1—H1A	109.5	N3—C10—H10A	109.5
N1—C1—H1B	109.5	N3—C10—H10B	109.5
H1A—C1—H1B	109.5	H10A—C10—H10B	109.5
N1—C1—H1C	109.5	N3—C10—H10C	109.5
H1A—C1—H1C	109.5	H10A—C10—H10C	109.5
H1B—C1—H1C	109.5	H10B—C10—H10C	109.5
O1—C2—O2	123.5 (2)	O3—C11—O4	122.9 (2)
O1—C2—C3	121.3 (2)	O3—C11—C12	117.7 (2)
O2—C2—C3	115.1 (2)	O4—C11—C12	119.3 (2)
N2—C3—C4	111.7 (2)	N4—C12—C13	111.2 (2)
N2—C3—C2	119.6 (2)	N4—C12—C11	120.8 (2)
C4—C3—C2	128.6 (2)	C13—C12—C11	128.0 (2)
C6—C4—C3	137.0 (2)	C14—C13—C18	119.8 (2)
C6—C4—C5	119.3 (2)	C14—C13—C12	103.7 (2)
C3—C4—C5	103.7 (2)	C18—C13—C12	136.5 (3)
N1—C5—C9	132.3 (3)	N3—C14—C13	106.8 (2)
N1—C5—C4	106.6 (2)	N3—C14—C15	130.8 (3)
C9—C5—C4	121.1 (3)	C13—C14—C15	122.3 (3)
C7—C6—C4	118.6 (3)	C16—C15—C14	117.2 (3)
C7—C6—H6A	120.7	C16—C15—H15A	121.4
C4—C6—H6A	120.7	C14—C15—H15A	121.4
C6—C7—C8	121.9 (3)	C15—C16—C17	121.8 (3)
C6—C7—H7A	119.1	C15—C16—H16A	119.1
C8—C7—H7A	119.1	C17—C16—H16A	119.1
C9—C8—C7	121.0 (3)	C18—C17—C16	121.5 (3)
C9—C8—H8A	119.5	C18—C17—H17A	119.2
C7—C8—H8A	119.5	C16—C17—H17A	119.2
C8—C9—C5	118.1 (3)	C17—C18—C13	117.3 (3)
C8—C9—H9A	121.0	C17—C18—H18A	121.3
C5—C9—H9A	121.0	C13—C18—H18A	121.3
			
C5—N1—N2—C3	1.9 (3)	C14—N3—N4—C12	−1.7 (3)
C1—N1—N2—C3	179.4 (3)	C10—N3—N4—C12	177.1 (2)
N1—N2—C3—C4	−2.2 (3)	N3—N4—C12—C13	0.8 (3)
N1—N2—C3—C2	−179.6 (2)	N3—N4—C12—C11	179.9 (2)
O1—C2—C3—N2	−4.2 (4)	O3—C11—C12—N4	175.5 (2)
O2—C2—C3—N2	178.0 (2)	O4—C11—C12—N4	−2.5 (4)
O1—C2—C3—C4	179.0 (3)	O3—C11—C12—C13	−5.5 (4)
O2—C2—C3—C4	1.1 (4)	O4—C11—C12—C13	176.5 (3)
N2—C3—C4—C6	−178.1 (3)	N4—C12—C13—C14	0.4 (3)
C2—C3—C4—C6	−1.0 (5)	C11—C12—C13—C14	−178.7 (3)
N2—C3—C4—C5	1.6 (3)	N4—C12—C13—C18	178.5 (3)
C2—C3—C4—C5	178.7 (3)	C11—C12—C13—C18	−0.6 (5)
N2—N1—C5—C9	178.6 (3)	N4—N3—C14—C13	2.0 (3)
C1—N1—C5—C9	1.4 (5)	C10—N3—C14—C13	−176.7 (3)
N2—N1—C5—C4	−1.0 (3)	N4—N3—C14—C15	179.1 (3)
C1—N1—C5—C4	−178.2 (3)	C10—N3—C14—C15	0.4 (5)
C6—C4—C5—N1	179.4 (2)	C18—C13—C14—N3	−179.9 (2)
C3—C4—C5—N1	−0.4 (3)	C12—C13—C14—N3	−1.4 (3)
C6—C4—C5—C9	−0.3 (4)	C18—C13—C14—C15	2.7 (4)
C3—C4—C5—C9	179.9 (2)	C12—C13—C14—C15	−178.8 (3)
C3—C4—C6—C7	179.1 (3)	N3—C14—C15—C16	−179.3 (3)
C5—C4—C6—C7	−0.5 (4)	C13—C14—C15—C16	−2.6 (4)
C4—C6—C7—C8	1.2 (4)	C14—C15—C16—C17	1.6 (5)
C6—C7—C8—C9	−1.1 (5)	C15—C16—C17—C18	−0.9 (5)
C7—C8—C9—C5	0.3 (5)	C16—C17—C18—C13	0.9 (5)
N1—C5—C9—C8	−179.2 (3)	C14—C13—C18—C17	−1.8 (4)
C4—C5—C9—C8	0.4 (4)	C12—C13—C18—C17	−179.7 (3)

### Hydrogen-bond geometry (Å, °)

**Table:**

*D*—H···*A*	*D*—H	H···*A*	*D*···*A*	*D*—H···*A*
O2—H2A···O1^i^	0.82	1.82	2.632 (3)	173
O3—H3A···O4^ii^	0.82	1.82	2.619 (3)	164
C8—H8A···O1^iii^	0.93	2.52	3.293 (4)	140

Symmetry codes: (i) −*x*, −*y*+1, −*z*+1; (ii) −*x*+1, −*y*+1, −*z*+1; (iii) *x*+1/2, −*y*+1/2, *z*−1/2.
